# Management of the Odontogenic Keratocyst – Six Cases with Conservative Management Supported by Chemical and Electrochemical Cauterization

**DOI:** 10.7759/cureus.6260

**Published:** 2019-11-30

**Authors:** Sathyabama Vijayarangan, Vikraman Baskara Pandian

**Affiliations:** 1 Oral & Maxillofacial Surgery, Ragas Dental College and Hospital, Chennai, IND

**Keywords:** conservative management of okc, parakeratinized, orthokeratinized, decompression, carnoy’s solution, odontogenic keratocyst, 3d reconstruction using "mimics" materialise software

## Abstract

Odontogenic keratocyst (OKC) has a special mention in the field of oral and maxillofacial surgery due to its varied presentation and high recurrence rate. The presence of Bcl-2 and cytokeratin 10 along with interleukins in the basal and suprabasal layers led to the inhibition of apoptosis of the surface epithelium and hence the high rate of recurrence. We discuss six cases diagnosed as odontogenic keratocyst on biopsy that underwent surgical removal. At the time of biopsy, the contents of the cyst were drained to allow decompression, then enucleation with Carnoy’s solution was performed as a secondary procedure. Based on our findings, we suggest a modification to Pogrel’s protocol of decompression with a drain in place followed by enucleation.

## Introduction

Treatment of odontogenic keratocyst (OKC) is one of the highly controversial protocols among oral and maxillofacial surgeons. Treatment modalities range from simple enucleation in the case of lesions that are less than 1 cm to extensive resection in the case of cysts that extend into the skeletal base. We discuss cystic cases that were diagnosed by biopsy with decompression followed by enucleation with the application of the Carnoy’s solution and/or electrochemical cauterization.

It was Broca who brought about the classification of odontogenic tumors in 1869, which was in use until 1914. Until the end of 1914, Broca’s classification of odontoma was maintained [1]. Later, in 1946, Thoma and Goldman classified odontogenic tumors into either ectodermal, mesodermal, or mixed origin [2]. In 1971, the first World Health Organization (WHO) classification of the tumors saw the representation of the cystic lesions [3]. Though this was maintained through 1992, Philipsen and Reichart reclassified them as tumors and the WHO named it as keratocystic odontogenic tumor in 2005, owing to the large rate of recurrence [4]. However, in 2017, the WHO reclassified odontogenic keratocyst back into the cystic category [5]. The aggressive behavior of the cyst has always puzzled surgeons as its expansion is based on the osmotic tension exerted on the adjacent tissues along with the inherent forces from within the epithelium or due to the enzymatic activity from the fibrous wall [6]. The expression of Bcl-2 and cytokeratin 10 in the suprabasal and basal cell layer brings about the anti-apoptotic property with considerable mitotic divisions resulting in increased survival of epithelial cells [7].

The aggressive nature of the cyst with its parakeratinized epithelium is dealt using decompression followed by enucleation along with the involved overlying mucosa [8,9]; this acts in addition to the application of Carnoy’s solution.

## Case presentation

Herein six cases of odontogenic keratocyst are presented. Each had extensive lesions due to their anterior-posterior expansion but were treated conservatively. A biopsy was done to confirm the diagnosis and to decompress the lesion, allowing the thickening of the lining epithelium to take place. Following this, the lesion was enucleated, and the lining epithelial fragments were meticulously removed from the cystic cavity along with the overlying mucosa. These areas were then subjected to cauterization with either Carnoy’s solution or electronically.

A 19-year-old woman presented with a concern of continuous mild gnawing pain over the left angle of the mandible for the past two weeks, with progressive trismus limiting her to a 26-mm mouth opening as well as signs of inferior alveolar nerve paresthesia. An orthopantomogram (OPG) followed by a computerized tomography (CT) scan confirmed the extent of the lesion.

**Figure 1:**
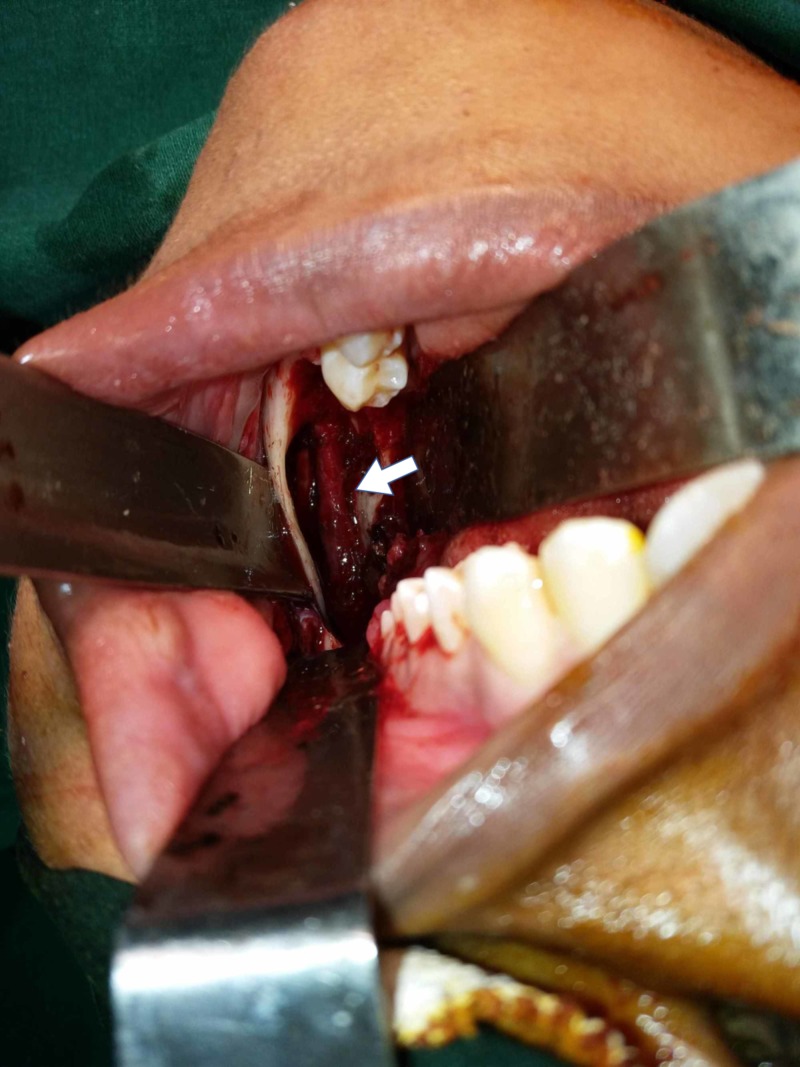
Enucleation of the cyst followed by lateralization of the inferior alveolar nerve with Carnoy’s solution

The aspirate was a cheese-like creamy material. A biopsy with decompression was performed. Odontogenic keratocyst was the histopathological diagnosis, and the patient was advised to have the cyst enucleated under general anesthesia. Henceforth, under general anesthesia, the cystic cavity was completely enucleated, the inferior alveolar nerve was lateralized (Figure 1), and the cavity was cauterized with the application of Carnoy’s solution for not more than two minutes. The bony margin forming the opening for the cystic cavity along with its attached overlying mucosa was removed, and the surgical site was closed primarily.

The second patient was a 39-year-old with a concern of mild pain and swelling that seemed to increase in size along the lower left posterior teeth region extending up to the mental foramen. As the latter was involved, the patient exhibited left mental nerve paresthesia.

**Figure 2:**
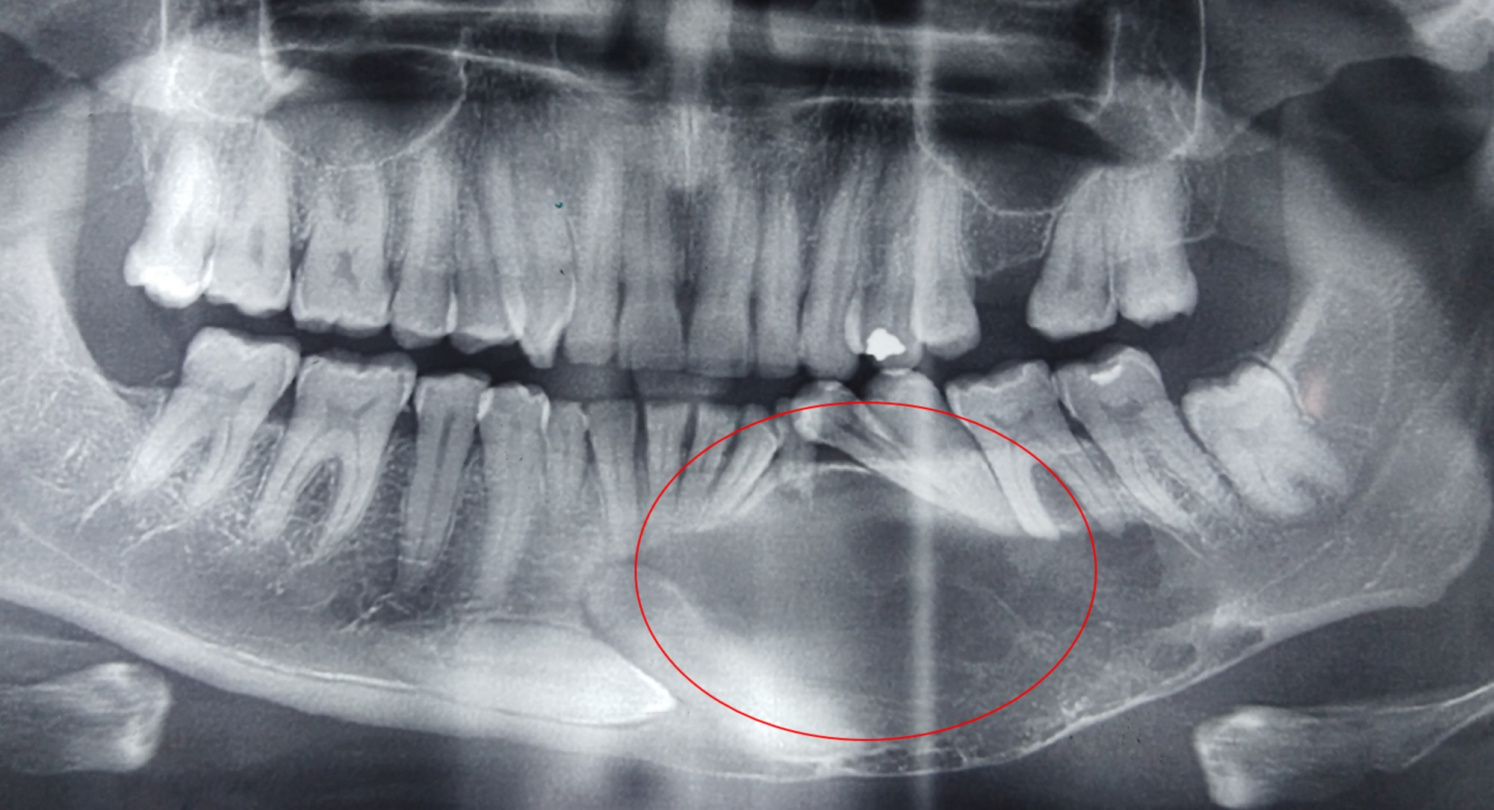
Orthopantomogram including the lesion and the impacted bilateral canines

An OPG revealed a huge cystic lesion extending from the first premolar on the right to the distal aspect of the second molar on the left side with impactions of both the canines at the symphyseal region (Figure 2). A cone-beam computed tomography scan was performed to correctly assess the location of the impacted canines and the relation of the cyst to the mental foramen and the impacted teeth.

An incisional biopsy was performed at the apical region of 34, where the cortical bone was soft in consistency and fluctuant which came back as orthokeratinized OKC. Under general anesthesia (GA), the cystic lesion was enucleated and the bony defect was filled with alloplastic bone.

The third patient was a 35-year-old woman with a concern of mild pain and sensitivity of the teeth in the maxillary anterior region. A CT was taken following the OPG to determine the involvement of teeth. An expansile lesion with bone loss extended from the first premolar on the left to that on the right. Mild infraorbital paresthesia of the left side was noted. Aspiration revealed a thin, straw-colored fluid, with a creamy, cheese-like material. Enucleation was performed under general anesthesia and the cavity was cauterized with electrotherapy. The bony defect was then grafted with autogenous bone from the iliac crest.

The fourth patient was a 29-year-old with a concern of halitosis and fluid leaking intraorally from the right body of the mandible. He had a horizontal impaction surgically removed by a local dentist. OPG revealed a well-scalloped radiolucent lesion extending from the mesial aspect of the first molar to the mandibular foramen that had become secondarily infected. Biopsy revealed parakeratinized odontogenic keratocyst that was planned for enucleation with chemical cauterization under general anesthesia with open dressing and debridement carried out meticulously as an outpatient. He was given upper and lower arch bars for elastics and intermaxillary fixation (IMF) during the first week of the post-operative period as the lesion had extended to the lower border, undermining it.

**Figure 3:**
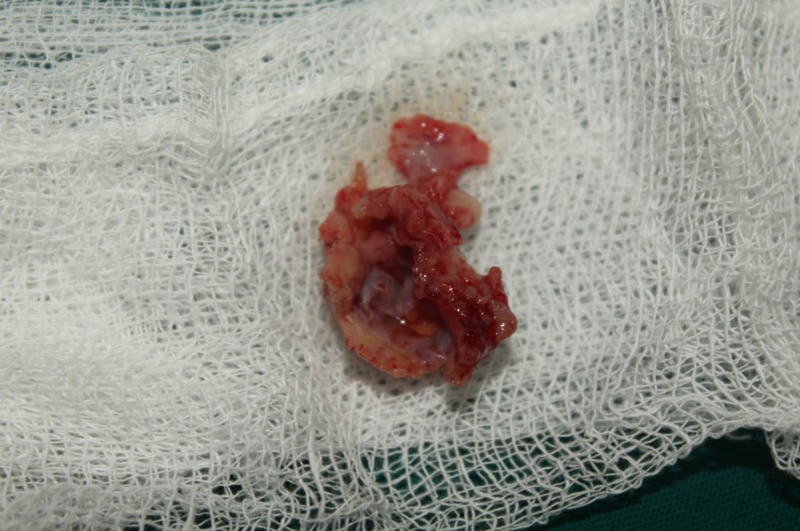
Excision of the overlying mucosa with the lesion

The fifth patient was a 35-year-old with an ongoing concern of pain in the 48 region for the past two weeks. On examination, she had an impacted tooth with a cystic lesion extending from the distal aspect of the first molar to the angle of the mandible involving the inferior third of the ramus. The biopsy specimen came back as orthokeratinized odontogenic keratocyst. Therefore, at that time in 2005, meticulous precaution was taken to ensure the cystic lining was not left behind, and the enucleation was carried out in totality along with the involved overlying mucosa (Figure 3). The cavity was packed with Carnoy’s solution and left for a couple of minutes before proceeding with the primary closure.

**Figure 4:**
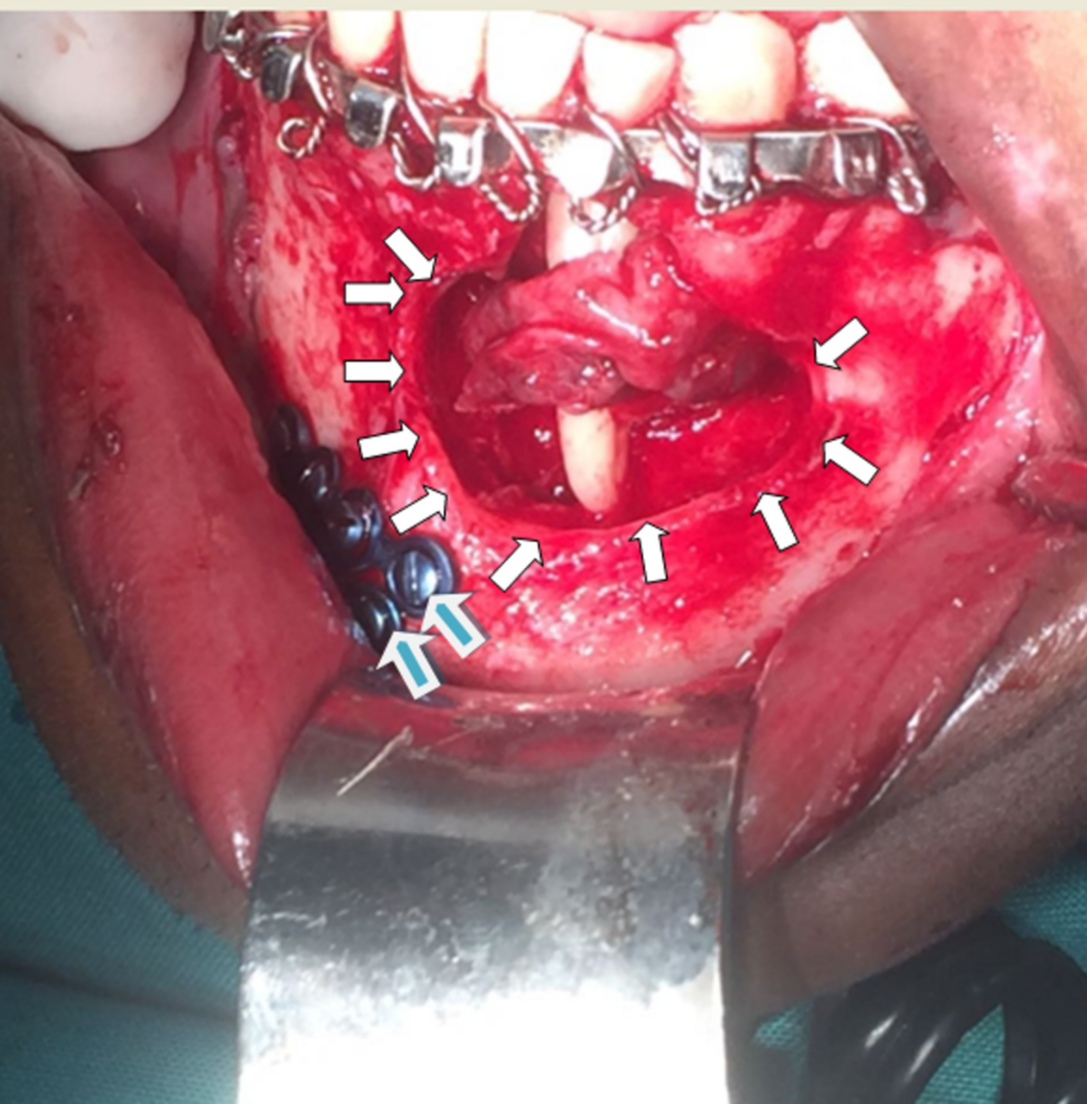
Mandibular fracture with cystic lesion The white arrows encircle the cystic lesion while the blue-colored arrows show the titanium plates used across the fracture.

The sixth patient was referred to the department for a concern of a mandibular symphyseal fracture and was found to have a cystic lesion in the anterior mandible. The lesion was curetted along with the fracture reduction (Figure 4). Test results of the cystic lesion came back as odontogenic keratocyst.

The cases discussed are provided in a tabulated format (Table 1).

**Table 1:** Tabulated format of the cases discussed RCT: Root canal therapy; OKC: Odontogenic keratocyst; PRF: Platelet-rich fibrin.

S No	Presenting Complaint	CL Presentation	Buccal and Lingual Expansion	Biopsy	Treatment	Carnoy	Bone Graft
1	Pain in L angle	Trismus with Inf alveolar paraesthesia	Present with trismus	Orthokeratinised OKC	Enucleation with curettage	Yes	PRF
2	Pain in L body up to mental foramen	Left mental nerve paraesthesia	Bilateral canine impaction at symphysis	Orthokeratinised OKC	Enucleation with curettage impaction removal	Yes	Allogenous bone
3	Sensitivity of upper anterior teeth	Soft labial swelling from premolar to premolar	Expansile lesion	Orthokeratin OKC	Enucleation with RCT of involved teeth	No	Iliac bone graft
4	Halitosis with fluid leaking	Sinus opening with secondary infection	Scalloped lesion from 1^st^ molar to mandibular foramen	Parakeratinised OKC	Open dressing with enucleation	Yes	Secondary healing
5	Pericoronitis – 48	Pain & swelling 48	Lesion from angle to the distal of 1^st^ molar	Orthokeratin OKC	Enucleation with primary closure	Yes	-
6	Fracture of symphysis	Displaced fracture with cystic lesion	Lesion of the mandible	Orthokeratin OKC	Lesion enucleated	Yes	-

## Discussion

All six cases involved the mandible except for the one case that involved the upper anterior premaxilla region. The lesions had generally obliterated the vestibule expanding both the buccal and the lingual cortical plates.

**Figure 5:**
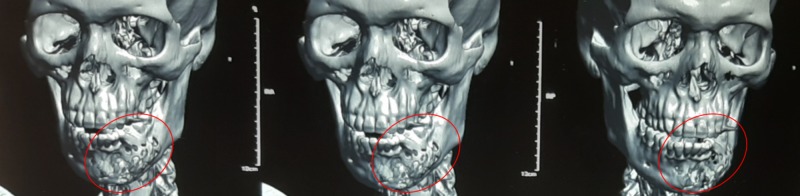
Three-dimensional reconstruction of the extent of the lesion using “MIMICS” - materialise software

The OPGs revealed well-defined radiolucent lesions with a scalloped margin in two cases. CTs with 3D reconstruction were taken to study the relations of the involved roots with the cyst (Figure 5). Perforations of the buccal and lingual cortices were noted in three cases; one showed a discontinuity of the lower border that, when explored intraoperatively, had an intact lower border.

**Figure 6:**
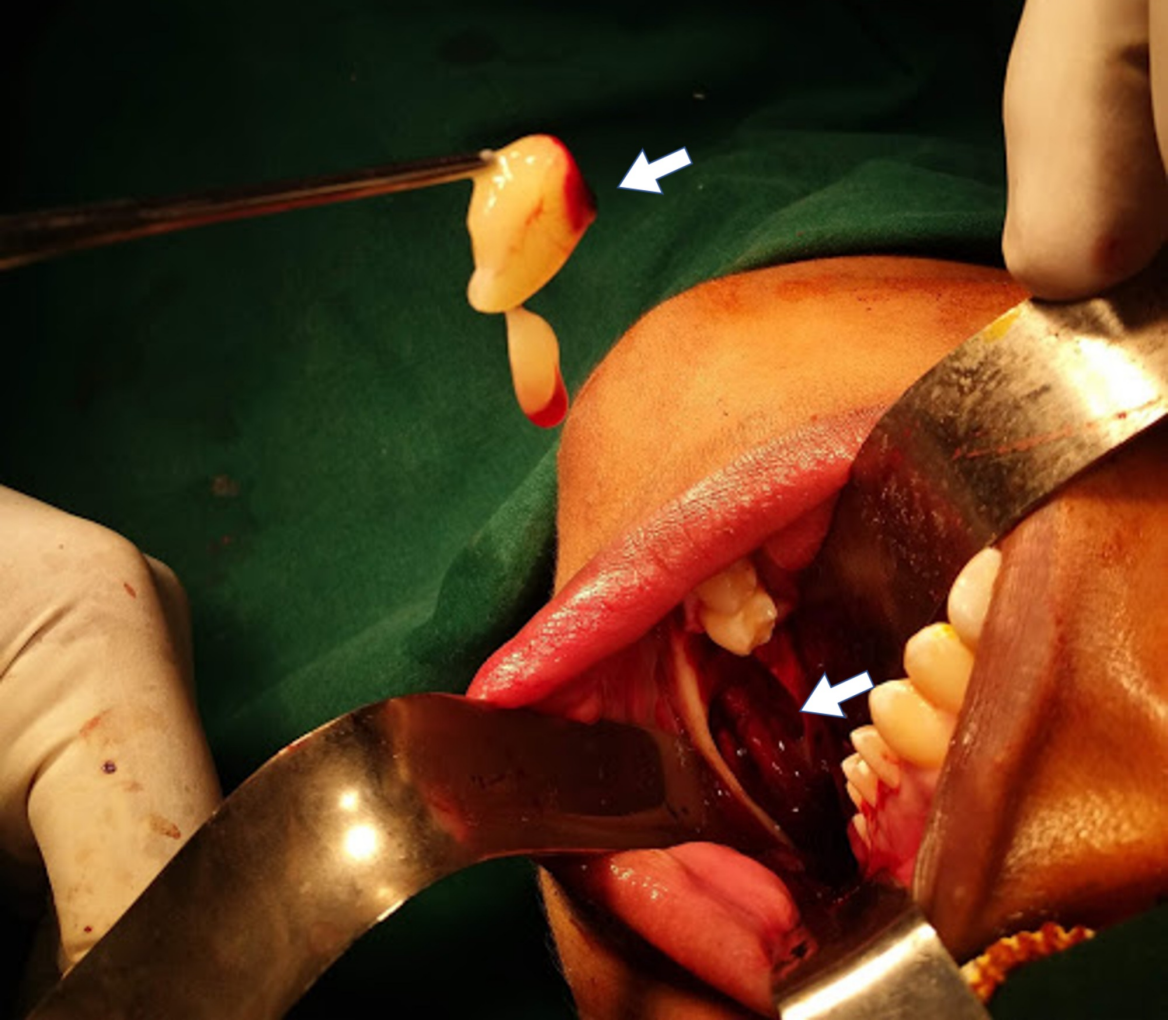
Cystic cavity grafted with allogenous bone and plasma rich fibrin

All the lesions when aspirated contained a creamy cheese-like material except for the one in the premaxilla region that instead contained a straw-colored fluid. The biopsy, however, confirmed that it was an odontogenic keratocyst. Four patients presented with the cyst having a sinus opening, and three were discharging fluid. The intraoral sinus seemed always to be present to the most mesial region of the lesion, leaving the ramus and the angle of the mandible intact. The biopsy was normally performed close to the sinus opening allowing the drainage of the lesion and was closed primarily after enucleation with alloplastic or autogenous bone graft with plasma rich fibrin (Figure 6).

**Figure 7:**
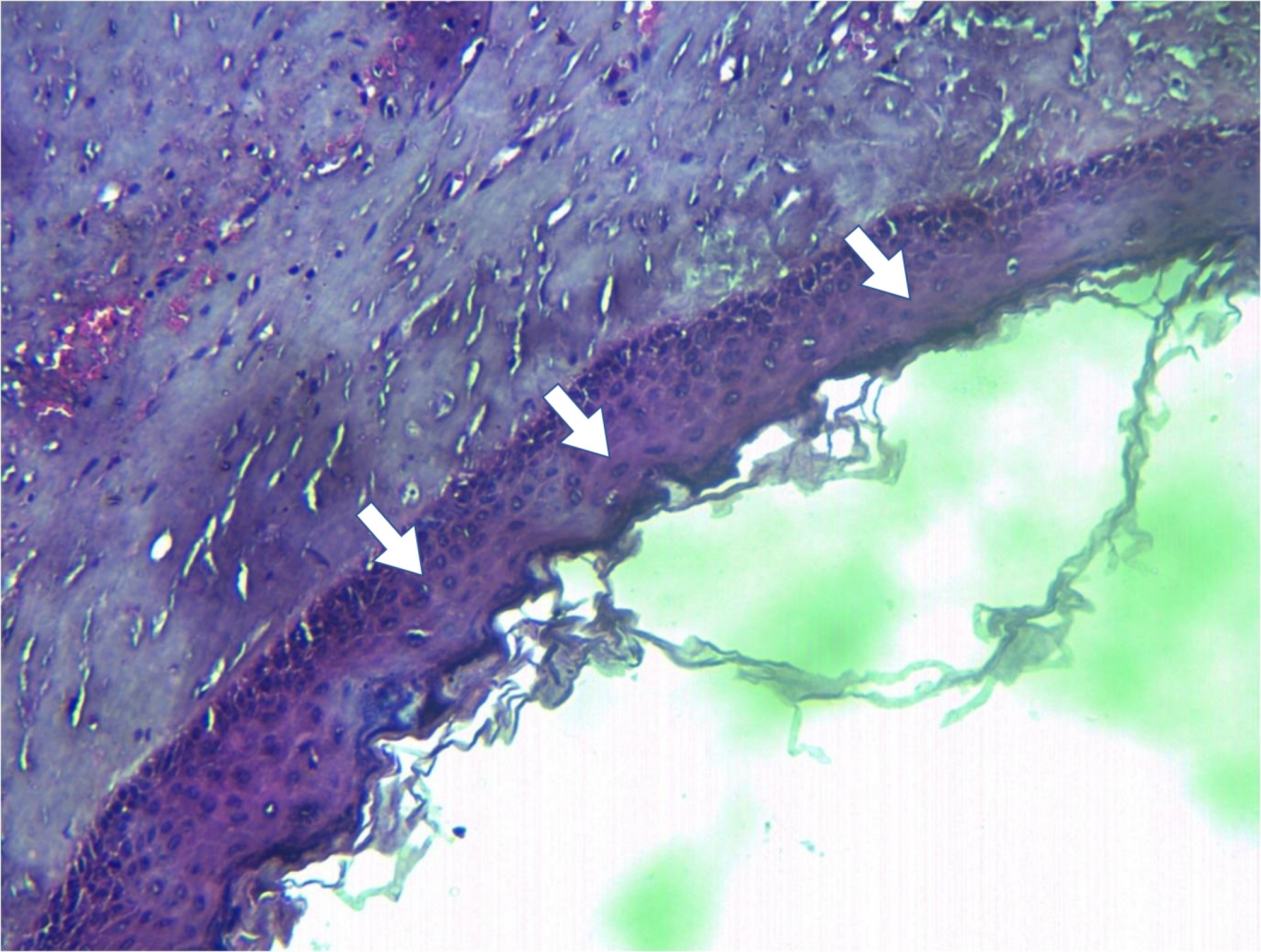
Photomicrograph of the epithelial lining of the odontogenic keratocyst

The biopsy reports of most cases came back as orthokeratinized (Figure 7), except for one at the angle of the mandible that was parakeratinized and was subjected to electrochemical cauterization. The aggressive nature of the parakeratinized lining as described by Shear [10] and Toller [11] was attributed to matrix metalloproteins and the interleukin-1 alpha bringing about the mural expansion within the basal and the suprabasal layers.

Under general anesthesia, a complete mucoperiosteal flap was elevated in the respective regions. The point of entry was always close to the biopsied site. Different surgeons were involved in the surgical procedures but all followed the same protocol of meticulous enucleation of the cystic lining along with the involved overlying mucosa, with lateralization of the inferior alveolar nerve when essential, along with Carnoy’s solution [12, 13]. The fifth case had to have his mental nerve avulsed along with the enucleation as the nerve was completely encapsulated within the cystic lesion.

**Figure 8:**
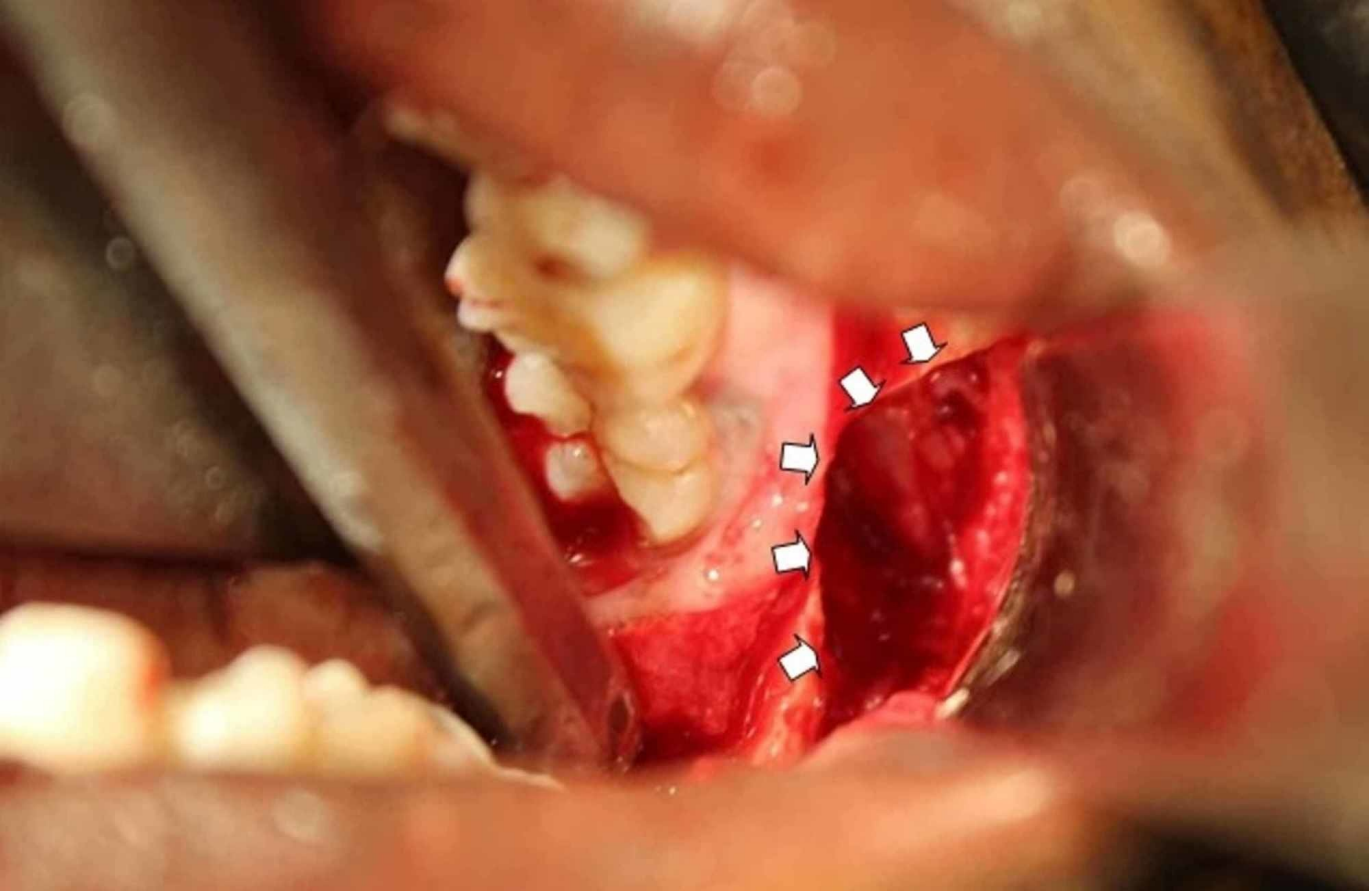
Lateral bony window created for access

In cases where the lower mandibular border was thinned out, precautionary measures were taken to protect against pathological fractures. One patient had an arch bar and the other an eyelet with IMF for three weeks. In the other, a lateral bony window was created (Figure 8) to help in enucleation. Five cases were closed primarily except the fourth patient who had an open dressing given and the cavity being allowed to heal secondarily. He however had implant-supported posterior teeth fabricated two years postoperatively.

The treatment modality varies from region to region. Huge lesions, especially the parakeratotic types, are dealt aggressively with enucleation and resection. The aggressive nature of OKC has been attributed not only to the presence of matrix metalloproteins as well as interleukin-1 alpha and parathyroid hormone-related protein but also to the presence of Bcl-2 and cytokeratin 10, leading to the high rate of recurrence [14].

The increase in morbidity associated with the resection and the compromise in the quality of life in dealing with large lesions has led surgeons to consider decompression followed by enucleation as an alternative treatment option. Pogrel reported a review of 10 cases of OKC that were treated conservatively with decompression and enucleation with no recurrence [15]. In his study, Voorsmit has proven that marsupialization followed by enucleation has no significant recurrence rate. Furthermore, Voorsmit et al. in their 1981 study, established that enucleation with Carnoy’s solution has a recurrence rate as low as 2.7% compared to 13.5% for an isolated enucleation [16]. It was Stoelinga who insisted that the removal of the overlying alveolar mucosa that forms the roof of the cystic cavity eliminates the presence of daughter cysts between the cyst lining and the alveolar mucosa [17].

The complete decompression of the cystic cavity by using a drain tube fixed to the first molar for eight to nine months, followed by a cystectomy done as a second stage procedure, was investigated by Brøndum and Jensen in 1991 [18]. Over an observation period of seven to 17 years, they found a 0% recurrence rate for a total of 12 keratocysts. Dammer et al. in their review of 52 cases, gave a verdict that smaller cysts that are less than 1 cm in dimension could be enucleated but the others that have invaded the adjacent soft tissue require radical treatment [19]. However, within the south Indian population, complete enucleation with either chemical or electronic cauterization has found no recurrence over a review period of 36 to 84 months in our study.

## Conclusions

Protocols should be in place that require dentists to perform a diagnostic OPG for patients on their first visit followed by imaging being done once every three years for a given individual so that these lesions could be discovered at an early stage. As for treatment options, a biopsy should be performed with the complete aspiration of the fluid contents that allows the thickening of the epithelium. The final treatment would be a conservative enucleation instead of an aggressive resection that mutilates patients, as most of these patients belong to the younger generation. None of these suggestions take away the fact that long-term follow-ups are essential with periodic radiographic examination.
